# Molecularly determined total tumour load in lymph nodes of stage I–II colon cancer patients correlates with high-risk factors. A multicentre prospective study

**DOI:** 10.1007/s00428-016-1990-1

**Published:** 2016-07-22

**Authors:** Iban Aldecoa, Begoña Atares, Jordi Tarragona, Laia Bernet, Jose Domingo Sardon, Teresa Pereda, Carlos Villar, M. Carmen Mendez, Elvira Gonzalez-Obeso, Kepa Elorriaga, Guadalupe Lopez Alonso, Javier Zamora, Nuria Planell, Jose Palacios, Antoni Castells, Xavier Matias-Guiu, Miriam Cuatrecasas

**Affiliations:** 1 Pathology Department, Centre de Diagnòstic Biomèdic (CDB), Hospital Clínic, University of Barcelona, Escala 3, Planta 5. Villarroel 170, Barcelona, 08036 Spain; 2 Pathology Department, Alava University Hospital, Vitoria-Gasteiz, Spain; 3 Pathology Department, Hospital Arnau de Vilanova, Lleida, Spain; 4 Pathology Department, Hospital L. Alcanyis, Xativa, Spain; 5 Surgery Department, Alava University Hospital, Txagorritxu, Spain; 6 Pathology Department, Hospital Costa del Sol, Marbella, Spain; 7 Pathology Department, Hospital Reina Sofia, Cordoba, Spain; 8 Pathology Department, Hospital Severo Ochoa, Leganes, Madrid, Spain; 9 Pathology Department, Hospital Onkologikoa, San Sebastian, Spain; 10 Pathology Department, Hospital 12 Octubre, Madrid, Spain; 11 Biostatistic Unit, Hospital Ramon y Cajal, Madrid, Spain; 12 Gastroenterology Department and Bioinformatics Unit, CIBERehd, IDIBAPS, Hospital Clinic, University of Barcelona, Barcelona, Spain; 13 Pathology Department, Hospital Ramon y Cajal, Madrid, Spain; 14 Gastroenterology Department, Hospital Clinic, University of Barcelona, IDIBAPS, CIBERehd, Barcelona, Spain; 15 CIBERehd, and Banc de Tumors-Biobanc Clinic-IDIBAPS-XBTC, Hospital Clinic, Barcelona, Spain

**Keywords:** Colorectal neoplasms, Neoplasm staging, Molecular pathology, Lymph nodes, Cytokeratin 19

## Abstract

Stage I–II (pN0) colorectal cancer patients are surgically treated although up to 25 % will eventually die from disease recurrence. Lymph node (LN) status is an independent prognostic factor in colorectal cancer (CRC), and molecular tumour detection in LN of early-stage CRC patients is associated with an increased risk of disease recurrence and poor survival. This prospective multicentre study aimed to determine the relationship between LN molecular tumour burden and conventional high-risk factors in stage I–II colon cancer patients. A total of 1940 LN from 149 pathologically assessed pN0 colon cancer patients were analysed for the amount of tumour cytokeratin 19 (CK19) messenger RNA (mRNA) with the quantitative reverse transcription loop-mediated isothermal amplification molecular assay One-Step Nucleic Acid Amplification. Patient’s total tumour load (TTL) resulted from the sum of all CK19 mRNA tumour copies/μL of each positive LN from the colectomy specimen. A median of 15 LN were procured per case (IQR 12;20). Molecular positivity correlated with high-grade (*p* < 0.01), mucinous/signet ring type (*p* = 0.017), male gender (*p* = 0.02), number of collected LN (*p* = 0.012) and total LN weight per case (*p* < 0.01). The TTL was related to pT stage (*p* = 0.01) and tumour size (*p* < 0.01) in low-grade tumours. Multivariate logistic regression showed independent correlation of molecular positivity with gender, tumour grade and number of fresh LN [AUC = 0.71 (95 % CI = 0.62–0.79)]. Our results show that lymph node CK19 mRNA detection correlates with classical high-risk factors in stage I–II colon cancer patients. Total tumour load is a quantitative and objective measure that may help to better stage early colon cancer patients.

**Electronic supplementary material:**

The online version of this article (doi:10.1007/s00428-016-1990-1) contains supplementary material, which is available to authorized users.

## Introduction

Surgical resection with no adjuvant therapy is recommended for most stage I–II colorectal cancer (CRC) patients, except for selected high-risk stage II patients given the significant impact of chemotherapy on stage III disease [1, 2]. Although there is evidence that pathological nodal staging is far from being optimal, current NCCN guidelines are based on haematoxylin and eosin (HE) lymph node (LN) staging, [3–7]. Its major weakness is the limited scope of histological LN analysis, based on a small sample provided by 2–5 μm LN sections, which comprise less than 0.5 % of the entire LN, and it may lead to false negative diagnoses [3, 7–9]. This may partly explain why up to 25 % of CRC patients with histologically negative LN die from recurrent disease after a potentially curative surgical resection. Some of these patients may have had undetected LN metastases [3, 10, 11].

The use of additional techniques, i.e. immunohistochemistry (IHC) or reverse transcriptase polymerase chain reaction, makes it possible to find LN tumour burden not detected with conventional HE analysis in 25 to 50 % of CRC patients, due to both increased sensitivity and the more extensive study than usually permitted by histological sections [3, 6, 7, 10–17].

Although the prognostic value of LN molecular tumour detection in early-stage CRC is controversial [14–18], there are enough data to support the use of more sensitive (i.e. molecular) methods of LN staging. As stated in three meta-analyses, the molecular detection of tumour cells in regional LN of stage I–II CRC patients is associated with an increased risk of disease recurrence and poor survival [3, 10, 11].

Most studies have focused in dichotomic (positive-negative) or semi-quantitative scales (isolated tumour cells (ITC), micro- and macrometastases) to assess molecular results [8, 10, 11, 14, 15, 17, 19, 20]. The molecular assay One-Step Nucleic Acid Amplification (OSNA; Sysmex Corporation, Kobe, Japan) is a quantitative method which analyses the entire LN. It amplifies cytokeratin 19 (CK19) mRNA from LN tissue lysates using the reverse transcription loop-mediated isothermal amplification (RT-LAMP) method [21]. CK19 mRNA was selected among other CRC markers showing the highest diagnostic performance and reproducibility, with 94.9–95.2 % sensitivity and 97.7–97.9 % specificity [22]. The system has been validated for breast and colon cancer LN assessment, providing results comparable to extensive histological and IHC LN analysis [8, 9, 21, 23–28]. The amount of CK19 mRNA detected correlates with the size of the metastatic foci [8, 9, 21, 27, 29]. It also makes it possible to calculate the total tumour load (TTL) present in a given patient, by adding all CK19 mRNA copies from each positive LN of a colectomy specimen [27, 28].

In this multicentre prospective study, we tried to correlate the TTL, as determined by OSNA, with classical clinical and pathologic high-risk factors, in an effort to determine whether the TTL could be used as an additional factor to better select stage I–II patients at risk of recurrence. Such an approach is now widely used in the treatment of breast cancer [27, 30, 31].

## Materials and methods

### Study sample

This is a prospective observational study including 10 institutions. Inclusion criteria were patients over 18 years old, with primary histologically confirmed colon cancer, cN0 preoperative diagnosis and positive CK19 IHC of the primary tumour. Exclusion criteria included rectal tumours, non-invasive pTis and pT0 tumours, positive LN on HE, synchronous tumours or other malignancies, cN1, gross adipose tissue involvement by the tumour, metastatic cancer, neo-adjuvant chemotherapy, familial adenomatous polyposis, carcinomas on inflammatory bowel disease and presence of stent-type intraluminal devices.

### Study procedure

#### Sample processing and fresh lymph node procuring

Fresh LN procurement from the mesocolon fat was performed within 50 min after surgical excision. When immediate LN dissection was not possible, the surgical specimen was kept up to 3 h in the refrigerator at 4 °C until LN dissection was done. During the LN harvesting process, the dissection area was kept cold by putting a thick layer of chopped ice under an elevated metallic surface and covered with a clean filter paper for LN dissection (Figure available at Online Resource 1a). Microcentrifuge tubes were also kept cold by punching them in chopped ice (Figure 2 available at Online Resource 1b). We first detached the mesocolon fat from the colon wall with a surgical blade. pT4 tumours corresponded to antimesenteric serous tumour infiltration, and pT3 tumours corresponded only to specimens with minimal tumour infiltration of the mesocolon. We then dissected one by one all LN from the mesocolon fat using different clean areas of the surgical blade for small LN, or changing it after each LN dissection. When a LN was grossly suspicious of being positive, a cytology touch-prep was performed to confirm or discard metastasis. Positive cases were discarded. All freshly dissected LN were analysed by both methods, HE and OSNA, using a modified protocol from previous studies [8, 9, 21]. Each LN was numbered and cut along the long axis. A central 1-mm slice was submitted for conventional formalin-fixation paraffin-embedding (FFPE) and HE analysis. The rest of the LN was stored at −80 °C in microcentrifuge tubes for 1 to 7 days until deferred OSNA analysis was performed. Lymph nodes with weight ≤0.07 g (average 5.5 mm) were defined as small.

After fresh LN harvesting, the specimen was fixed overnight in 10 % neutral buffered formalin. Then, the mesocolon fat was re-examined for remaining LNs, which were submitted only for conventional histopathology analysis.

#### CK19 immunohistochemistry

**Fig. 1:**
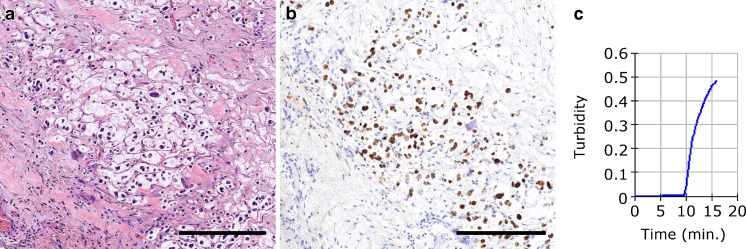
**a** A primary high-grade mucinous carcinoma with signet ring cells. **b** Positive CK19 immunohistochemistry from the same case. **c** Amplification curve of *CK19* mRNA from a positive LN, assessed through turbidity variation (*Y*-axis) over time (*X*-axis, minutes). (Fig. 1a, b, scale bar 250 μm)

CK19 IHC was performed on representative sections of all the primary colon carcinomas to ensure reliable negative molecular CK19 mRNA results. A 2-μm section of each primary tumour was mounted on FLEX IHC microscope slides and pre-treated in PT-LINK (Dako, Glostrup, Denmark). Incubation for 20 min with the primary CK19 antibody (CK19 mouse monoclonal, clone RCK108; IR615 pre-diluted. Dako) was performed in the AutostainerLink 48 (Dako). Membranous staining with or without cytoplasm staining of ≥10 % of the tumour cells was defined as positive IHC in colon carcinomas (Fig. 1a, b).

#### Pathology report and LN staging

LN staging and pathology report were performed from the analysis of HE stains according to the AJCC/UICC TNM, 7th edition [32, 33]. Tumours ≥4 cm were defined as large. Pathologists and clinicians were both blinded to the OSNA results.

#### OSNA procedure

The OSNA method was performed at each institution following the manufacturer’s instructions, using the protocol described by Tsujimoto et al. [21]. Briefly, LNs were weighed, homogenized with the lysis buffer Lynorhag (Sysmex) for mRNA stabilization and genomic DNA precipitation. After centrifugation for 1 min at 10,000 × *g* at room temperature; a 2-μL sample of the intermediate phase was mixed with the reagent LynoampBC (Sysmex). Analysis was performed using the RT-LAMP isothermal amplification method with the RD-100i automated gene-amplification system (Sysmex). The amount of CK19 mRNA amplified was detected by a change in turbidity upon precipitation of magnesium pyrophosphate (Fig. 1c). The result was correlated to CK19 mRNA copy number/μL of the original lysate through calibrated standard curves containing different CK19 mRNA concentrations. In every assay, a standard positive control sample containing 5 × 10^3^ copies/μL of CK19 mRNA, and a negative control sample without CK19 mRNA were used for validation. The results were based on the number of CK19 mRNA copies/μL obtained for each LN. The cutoff value was 250 copies/μL, based on a previous study [9].

### Statistical analysis

The Fisher exact test and Pearson’s correlation coefficient were used for testing the association between categorical or numerical variables, respectively. The Mann-Whitney-Wilcoxon and Kruskal-Wallis test were applied to compare groups’ distributions. A *p* < 0.05 was considered statistically significant.

Logistic regression analysis was used to predict the OSNA outcome. The backward stepwise algorithm was used to determine the best-fitting model. The classification avidity of the model was assessed by the ROC curve and a 10-fold cross-validation technique was applied for the model validation. The Cohen’s kappa was used to assess the degree of agreement between OSNA outcome and model prediction. All analyses were performed using *R* statistical environment (V.3.1.0) [34].

## Results

### Sample size and characteristics

**Fig. 2:**
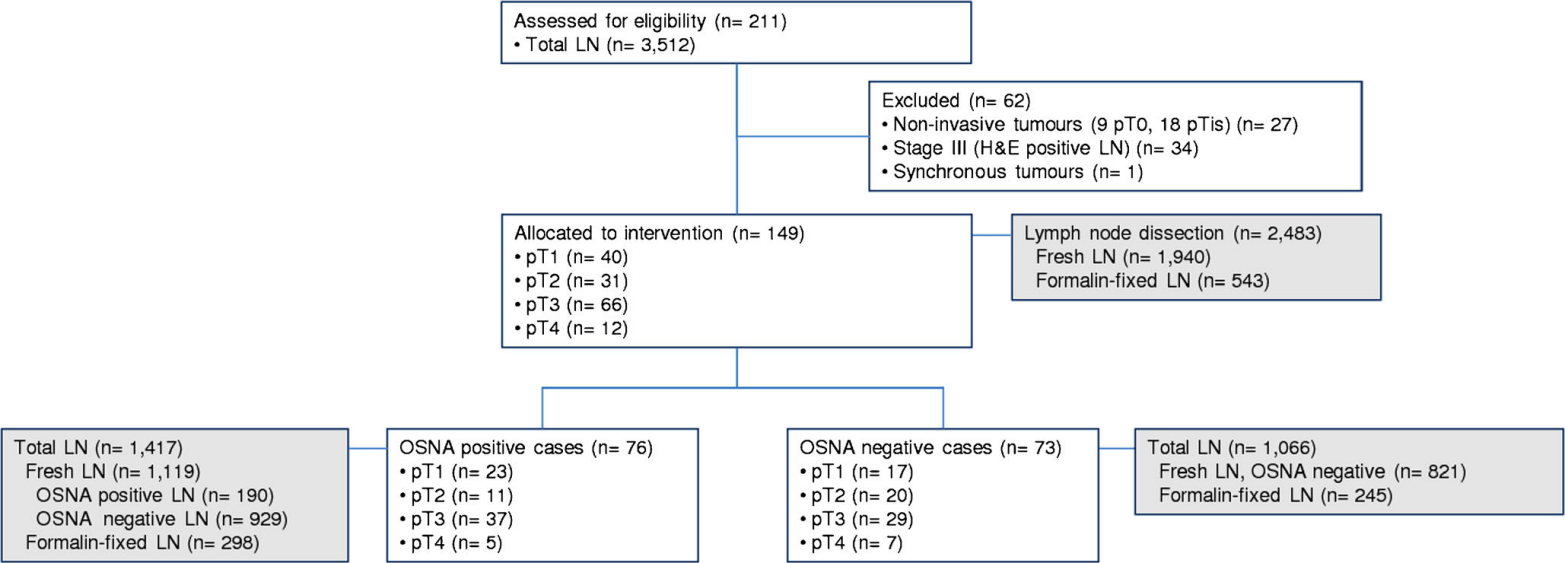
Study flow diagram

We analysed 3512 LN from 211 colon cancer patients recruited in 10 hospitals between June 2012 and December 2013. We excluded 27 non-invasive tumours (9 pT0 and 18 pTis), 34 cases with HE-positive LN, and one patient with synchronous tumours. Finally, 149 stage I–II colon cancer patients met the study selection criteria. All primary tumours showed positivity for CK19 IHC. Demographic, clinical, pathological, and lymph node characteristics of the study sample and correlation with CK19 mRNA results are summarized in Table 1 and the study flow diagram (Fig. 2).

**Table 1:** Patient’s demographics and specimen characteristics and correlation with CK19 mRNA results

Clinical parameter	*n* (%)	CK19 mRNA negative n (%)	CK19 mRNA positive n (%)	*p* value
Cases	149 (100)	73 (49.0)	76 (51.0)	–
Gender				0.02
Male	97 (65.1)	41 (42.3)	56 (57.7)	
Female	52 (34.9)	32 (61.5)	20 (38.5)	
Age (years)*—*median (IQR)	67 (61–75)	68 (61–74)	66 (61–75)	0.89
Surgical specimen characteristics				
Specimen size (cm)*—*median (IQR)	20 (15–25)	20 (15–25)	19.5 (15–25)	0.81
Tumour size (cm)^a^—median (IQR)	3 (2–5)	3 (2–4)	4 (2–5.5)	0.045
Large tumours (>4 cm)	39 (26.7)	14 (38.9)	25 (64.1)	0.09
Small tumours (≤4 cm)	107 (73.3)	57 (53.3)	50 (46.7)	
Tumour location				0.33
Right colon and caecum (incl. hepatic flexure)	67 (45)	37 (55.2)	30 (44.8)	
Transverse colon	14 (9.4)	5 (35.7)	9 (64.3)	
Left colon and sigmoid colon (incl. splenic flexure)	68 (45.6)	31 (45.6)	37 (54.4)	
Macroscopic tumour configuration				0.57
Annular	8 (5.4)	5 (62.5)	3 (37.5)	
Ulcerated	65 (43.6)	33 (50.8)	32 (49.2)	
Polypoid	75 (50.3)	34 (45.3)	41 (54.7)	
Other	1 (0.7)	1 (100)	0 (0)	
Vascular invasion				0.45
No	137 (91.9)	65 (47.4)	72 (52.6)	
Yes	12 (8.1)	8 (66.7)	4 (33.3)	
Histological type				0.017
Adenocarcinoma	136 (91.3)	71 (52.2)	65 (47.8)	
Mucinous/signet ring cell AC	13 (8.7)	2 (15.4)	11 (84.6)	
Grade^b^				<0.01
High grade	22 (15.0)	4 (18.2)	18 (81.8)	
Low grade	125 (85.0)	69 (55.2)	56 (44.8)	
pT				0.19
pT1	40 (26.8)	17 (42.5)	23 (57.5)	
pT2	31 (20.8)	20 (64.5)	11 (35.5)	
pT3	66 (44.3)	29 (43.9)	37 (56.1)	
pT4a	12 (8.1)	7 (58.3)	5 (41.7)	

*IQR* interquartile range, *AC* adenocarcinoma

^a^In three patients, tumour size could not be assessed

^b^In two patients, tumour grade could not be assessed

### Lymph node features and CK19 mRNA results

Among the 2483 LN procured from 149 cases, slightly over 78 % of them were freshly isolated and submitted for OSNA analysis (1940 fresh LN vs 543 FFPE LN). Hence, we obtained a median of 15 LN per case, of which 12 LN were analysed by OSNA and HE.

**Fig. 3:**
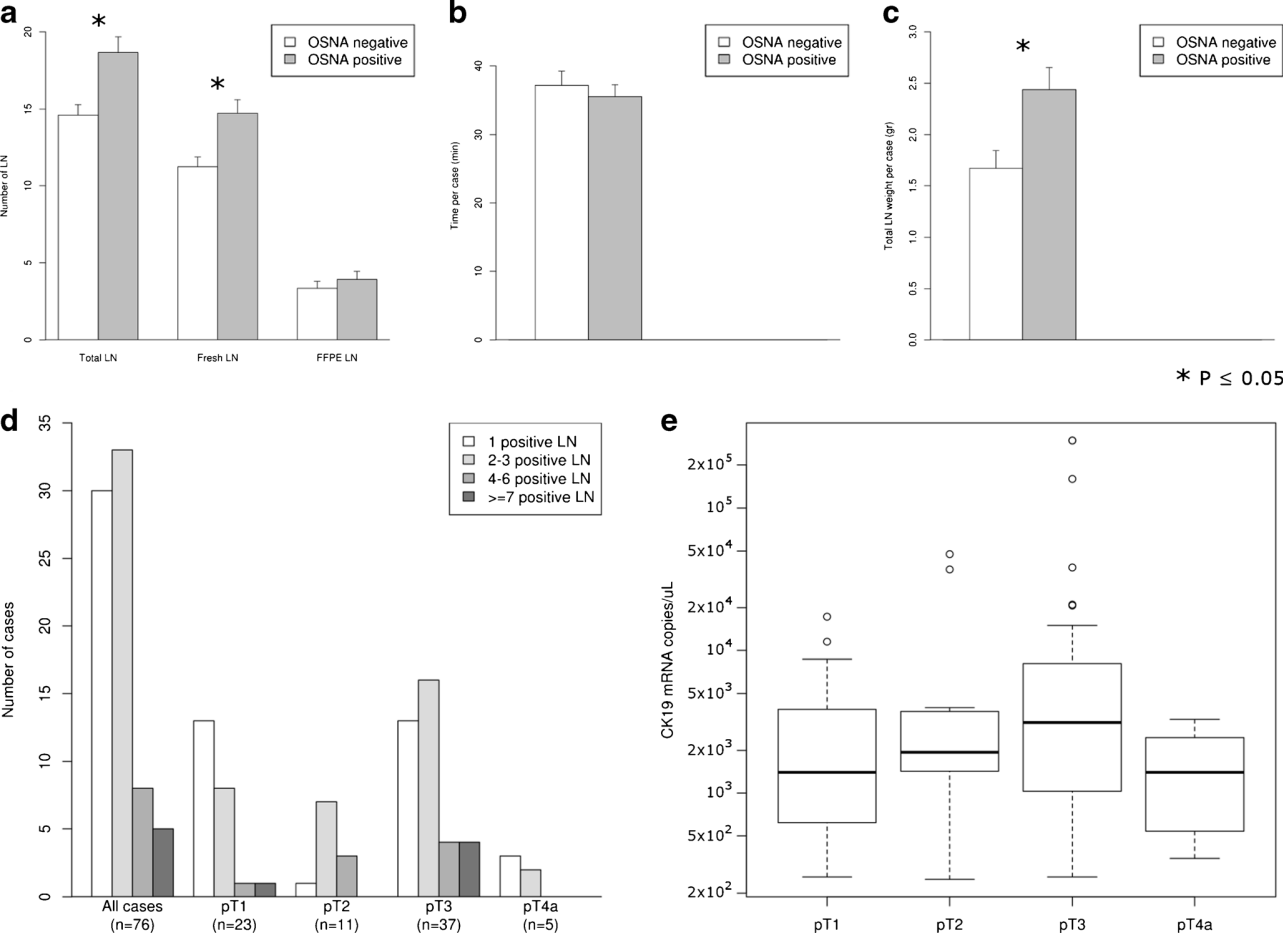
OSNA results and correlation with LN retrieval and pT stage, regarding number of LN retrieved (**a**), time spent on fresh LN search in minutes (**b**) and total weight of fresh LN per case (gr) (**c**). OSNA positive cases held significantly more LN due to a higher fresh LN yields. **d** Shows that most cases in every pT stage held up to 3 OSNA positive LN. **e** Although not significant, a trend was observed between pT stage and TTL

CK19 mRNA positivity was observed in 76/149 patients (51 %). Most of those positive cases; i.e. 80 %, had only 1 to 3 positive LN. Thus, among all OSNA LN analysed, 9,8 % (190/1940) were positive. Association analysis showed that OSNA-positive cases harboured more freshly procured LN (*p* = 0.012) and had a higher total LN weight per case (*p* < 0.01) than negative ones (Fig. 3). Regarding the size of the freshly dissected LN, CK19 mRNA-negative LN were significantly smaller (i.e. weight ≤0.07 g) than positive ones (29.3 vs 15.6 %, *p* < 0.01).

In conclusion, fresh LN dissection did not impede an effective LN dissection and was an efficient way to obtain the minimum number of LN required for pN assessment. Although a substantial part of cases harboured tumour burden, only a few LN were positive among them.

### High-risk factors and CK19 mRNA detection

The pathological high-risk features of CRC that were related to the presence of tumour CK19 mRNA in the LN were as follows: mucinous/signet ring histological types, high histological grade and tumour size. Among demographic variables, male gender was significantly related to OSNA positivity. No relationship was found with pT stage, vascular invasion, macroscopic tumour configuration, or other features.

High-grade tumours were significantly larger than their low-grade counterparts (*p* < 0.01), with a trend between histological type and tumour size (*p* = 0.11). The logistic regression model including OSNA results as dependent variable and tumour size as independent variable adjusted by histological type or grade showed a no significant relation between tumour size and OSNA response (*p* = 0.26 and *p* = 0.15, respectively).

**Fig. 4:**
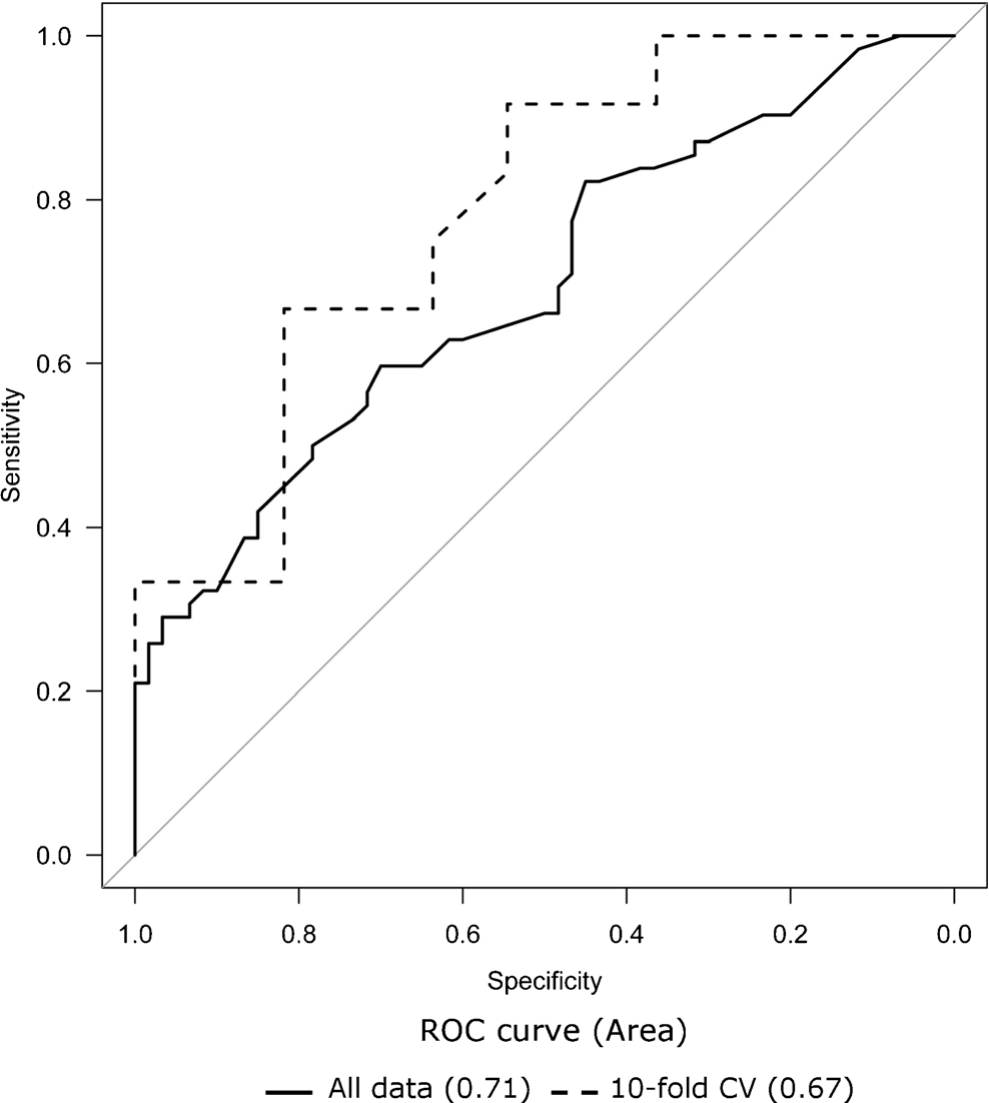
ROC curve of the multivariate logistic regression model for OSNA results including gender, grade and the number of fresh lymph nodes as variables predicting molecular positivity. *Continuous line*: All data (0.71 (95 % CI = 0.62–0.79); *Dashed line*: 10-fold CV (AUC = 0.67 [95 % CI = 0.59–0.76])

The multivariate logistic regression analysis gave a reduced predictive model for OSNA molecular output including the independent variables gender, grade, and the number of fresh lymph nodes (Table 2). The receiver operating characteristic (ROC) curve was performed to test the avidity of the generated model to predict CK19 mRNA outcome, giving an AUC = 0.71 (95 % CI = 0.62–0.79). With a decision boundary of 0.5, the sensitivity, specificity, and accuracy were 61, 68, and 64 %, respectively. A 10-fold cross-validation was done for the model assessment, the resulting AUC was 0.67 (95 % CI = 0.59–0.76) (Fig. 4). Therefore, male gender, high histological grade and higher yields of LN were independent predictors of CK19 mRNA positivity.

**Table 2:** Logistic regression of clinical and histological variables related to CK19 mRNA positivity

Variables	Univariate model		Multivariate model	
	OR (95 % CI)	Wald test *p*	OR (95 % CI)	Wald test *p*
Gender (male vs female)	2.2 (1.1–4.4)	0.026	3.1 (1.4–7.0)	0.006
Age (years)	0.99 (0.96–1.03)	0.74	–	–
Tumour size	1.2 (1.0–1.4)	0.06	–	–
Tumour location		0.31	–	–
Transverse colon vs right colon and caecum (including hepatic flexure)	2.2 (0.7–7.9)			
Left colon and sigmoid colon (including splenic flexure) vs Right colon and caecum (including hepatic flexure)	1.4 (0.7–2.9)			
Macroscopic tumour configuration		0.59	–	–
Ulcerated vs annular	1.6 (0.4–8.4)			
Polypoid vs annular	2.0 (0.5–10.4)			
Histological type (mucinous / signet ring cell AC vs adenocarcinoma)		0.02	–	–
	6.0 (1.5–39.8)			
Vascular invasion (yes vs no)	0.5 (0.1–1.5)	0.21	–	–
Grade (high vs low)	5.4 (1.9–19.5)	<0.01	4.8 (1.5–18.9)	0.013
pT		0.20	–	–
pT2 vs pT1	0.4 (0.2–1.1)			
pT3 vs pT1	0.9 (0.4–2.1)			
pT4a vs pT1	0.5 (0.1–1.9)			
Fresh LN per case weight (gr)	1.1 (1.0–1.1)	<0.01	1.1 (1.0–1.2)	0.017

*AC* adenocarcinoma, *gr* grammes

### Total tumour load

The median TTL of CK19 mRNA copies/μL among positive cases was 2015 (IQR: 940–6222.5). Cumulative TTL showed that 29 % of patients (22/76) had more than 5000 CK19 mRNA copies/μL. Although not significant (*p* = 0.18), there was a positive trend between CK19 mRNA copies/μL and pT stage; while TTL in pT1 cases was 1280 CK19 mRNA copies/μL and 1790 CK19 mRNA copies/μL in pT2, it rose to 3080 CK19 mRNA copies/μL in pT3 tumours (Fig. 3e). Surprisingly, the median TTL among pT4a tumours was 540 CK19 mRNA copies/μL. This could be explained by the inclusion of antimesenteric pT4 tumours rather than bulky tumours with marked adipose tissue infiltration, to the scarce amount of positive cases (5 among pT4a tumours), and by the fact than in 3 of them less than 65 % of the LNs were analysed by OSNA. Distribution of the cases regarding their TTLs and divided by pT stage is provided as Supplementary Table 1 in Online Resource 2. Albeit also not significant, all OSNA positive cases with vascular invasion had >2000 CK19 mRNA copies/μL.

In order to evaluate the distribution of the TTL, it was categorized by quartiles and compared with classical high-risk factors (data not shown). When stratified by tumour grade, it showed that among low-grade tumours, TTL increased with tumour stage (pT) (*p* = 0.01) and tumour size (*p* < 0.01; rho = 0.27) (Table 3). This trend was not observed in high-grade tumours. Taken together, our results show that most patients had low TTLs. These values increased with tumour stage and followed different distributions among low- and high-grade tumours.

**Table 3:** Total tumour load distribution in high and low-grade tumours

pT	CK19 mRNA copies/μL per case *n* (%)				Total *n* (%)	*p* value
	<1000	<2000	<6000	≥6000		
All cases						0.246
pT1	8 (34.8)^a^	6 (26.1)	6 (26.1)	3 (13.0)	23 (100)	
pT2	1 (9.1)	5 (45.4)	3 (27.3)	2 (18.2)	11 (100)	
pT3	9 (24.3)	6 (16.2)	8 (21.6)^a^	14 (37.8)	37 (100)	
pT4a	2 (40.0)	1 (20.0)	2 (40.0)	0 (0.0)	5 (100)	
Total	20 (26.3)	18 (23.7)	19 (25)	19 (25)	76 (100)	
High grade						0.61
pT1	1 (20.0)	1 (20.0)	2 (40.0)	1 (20.0)	5 (100)	
pT2	1 (50.0)	0 (0.0)	0 (0.0)	1 (50.0)	2 (100)	
pT3	1 (12.5)	4 (50.0)	1 (12.5)	2 (25.0)	8 (100)	
pT4a	1 (33.3)	0 (0.0)	2 (66.7)	0 (0.0)	3 (100)	
Total	4 (22.2)	5 (27.8)	5 (27.8)	4 (22.2)	18 (100)	
Low grade						0.01
pT1	6 (35.3)	5 (29.4)	4 (23.5)	2 (11.8)	17 (100)	
pT2	0 (0.0)	5 (55.6)	3 (33.3)	1 (11.1)	9 (100)	
pT3	8 (28.6)	2 (7.1)	6 (21.4)	12 (42.9)	28 (100)	
pT4a	1 (50.0)	1 (50.0)	0 (0.0)	0 (0.0)	2 (100)	
Total	15 (26.8)	13 (23.2)	13 (23.2)	15 (26.8)	56 (100)	

^a^In two patients, tumour grade could not be assessed

### Patient’s follow-up

At the time of writing of this manuscript, all patients had a follow-up of 2 years (median 33 months, IQR 25–32.5); 8.7 % (13/149) patients recurred (3 died and 10 were alive with metastatic disease). Four of the cases were OSNA positive, with a median TTL of 4375 CK19 mRNA copies/μL (range 360–47,600); 2 of the 3 dead patients were OSNA positive.

## Discussion

This study quantifies the amount of total tumour load within the lymph nodes of stage I–II colon cancer patients, and suggests that LN molecular detection of CK19 mRNA could become an objective indicator of risk in such individuals [11, 20, 28, 35]. Stage I–II CRC is amenable to complete surgical resection, but patients at risk of tumour recurrence are clinically and pathologically difficult to identify [36, 37]. In addition, cancer dissemination to LN may occur at early stages of tumour development [6, 38], potentially implying a poor prognostic impact [3, 7, 11, 15]. Thus, the assessment of the nodal staging arises as a key factor for CRC therapeutic management [3, 10, 11].

Our findings confirm the presence of undetected nodal tumour burden in early-stage colon cancer using the OSNA method. Compared to previous studies, we have the highest percentage of cases with CK19 mRNA detection in LN (i.e. 51 %) [23–25, 28]. Discrepancies may be due to differences in the LN collection and analysis, such as the fact that we analysed a larger amount of LN, including all evaluable LN by OSNA regardless of its size. Current guidelines recommend evaluating at least 12 LN to achieve a reliable histological staging [16, 32, 33, 39–42]. In compliance with them, we obtained a median of 15 LN per patient, of which 12 were freshly obtained and analysed by OSNA.

CRC lymph nodes are usually small, especially in early stages of the disease, but may still contain tumour burden, as size is not a good preoperative indicator of LN staging, or a predictor of the presence of tumour [43–45]. Although we identified a significant association between CK19 mRNA detection and larger LN size, we found CK19 mRNA in 15.6 % of small LN. Furthermore, the multivariate analysis of OSNA results showed that the number of collected LN, the gender and the histologic grade were independent predictors of OSNA results. Our data highlights the importance of procuring any identifiable LN irrespective of its size [40–42, 45–47].

Our results show that the presence of CK19 mRNA in regional LN was associated with other classical high-risk factors such as mucinous/signet ring types, histologic high-grade, tumour size and male gender. Moreover, it is noteworthy the trend observed between TTL and pT stage, as pT3 had the highest TTL (3080 CK19 mRNA copies/μL) and rate of CK19 mRNA detection (37/66).

We also observed a different behaviour between low and high-grade tumours, with a significant orderly increase of TTL in regional LN with the increase of pT stage and tumour size in low-grade tumours. This phenomenon may be accounted for by the fact that low-grade tumours tend to follow an orderly sequence of accumulation of molecular alterations as they grow and acquire infiltrative proprieties [48]. In contrast, no association was found with any assessed variables in high-grade tumours. This may be explained by the different molecular pathways involved in high-grade tumours and their aggressive behaviour from the beginning [48]. Reinforcing the importance of tumour grade in neoplastic LN spread, tumour size did not correlate with CK19 mRNA positivity when stratified by grade or histological type.

The biologic significance of LN molecular tumour detection is of leading importance. Although the sole presence of small amounts of tumour cells in regional LN does not necessarily imply poor prognosis, it may indicate a yet undefined risk of disease recurrence. This would explain why, with historical recurrence records of 2.5 % in pT1 N0 tumours, we have found that almost 60 % had some CK19 mRNA tumour burden in their LN [11, 49]. In this context, low TTLs may have no further biologic consequences, but large amounts of tumour burden within the LN may imply higher odds of recurrence [7, 27]. A study using RT-qPCR detection of Guanylyl cyclase C (GUCY2C) by RT-qPCR found 87.5 % patients with positive LN, although only 20.9 % developed recurrent disease [7].

Quantifying the amount of tumour burden, not just the presence or absence of tumour, in regional lymph nodes is therefore important in prognosticating from molecular results, as has been stated in CRC [3, 7, 11, 20, 35] and breast cancer [27, 31]. It should be highlighted that whereas the median TTL of our cohort was 2015 CK19 mRNA copies/μL, values up to 15,000 CK19 mRNA copies/μL have been recently set in breast cancer sentinel LN as clinically relevant to predict additional axillary LN metastases [27]. Some authors have also demonstrated the relationship between the amount of metastatic tumour and prognosis, stressing the importance of distinguishing ITC from micrometastases [7, 11, 23, 24, 50]. Two novel Japanese multicentre studies stress the need to stratify molecular outcomes in LN assessment. The first one addresses that TTL significantly increases with pN stage [28] and suggests it as a potential staging technique. The second one has shown the correlation of LN micrometastasis volume, measured by qRT-PCR for CEA mRNA, as a predictor of recurrence in stage II patients [51]. In addition to these findings, we have found that TTL unveils distinct progression patterns in low- and high-grade neoplasms.

Our study has some drawbacks; firstly, a follow-up period of 2 years is a limited time to establish the amount of CK19 mRNA copies that would have clinical significance; thus, the OSNA results could not be correlated with prognosis. Nevertheless, this study was not designed to determine the role of the molecular results as a prognostic factor, but to find out whether the OSNA results correlated with other classical CRC high-risk factors. Secondly, in order to evaluate the histological high-risk factors, we focused on histologically pN0 specimens. To assess the potential predictive value of the TTL, colectomy specimens should be studied regardless of their LN status, thus comparing histological and molecular evaluation methods and their ability to predict disease recurrence.

In conclusion, this study identifies the presence of undetected tumour burden in LN of early colon cancer patients. TTL correlates with other CRC classical high-risk factors and may be placed among them. We thereby support CK19 mRNA TTL as a fast and reliable approach to help better stage early colon cancer. Long-term follow-up and validation studies are needed to obtain a predictive prognostic scale for stage I–II colon cancer patients based on the patient’s TTL.

AC, adenocarcinoma; AUC, area under the curve; CK19, cytokeratin 19; CRC, colorectal cancer; FFPE, formalin-fixation paraffin-embedding; HE, Haematoxylin and Eosin; IHC, immunohistochemistry; IQR, interquartile range; ITC, isolated tumour cells; LN, lymph node; OSNA, one-step nucleic acid amplification; pT, tumour stage; ROC, receiver-operator characteristic; RT-LAMP, reverse transcription loop-mediated isothermal amplification; TTL, total tumour load

## Electronic Supplementary Material

ESM 1 Material used for fresh lymph node retrieval. (a) Image of the dissection area, with a thick layer of chopped ice beneath an elevated metallic surface. By placing filter paper above, the fresh LN dissection could be performed on a clean surface. (b) Microcentrifuge tubes were also kept cold by holding them in chopped ice (PDF 160 kb)

ESM 2 Case distribution regarding TTL values and pT stage. The distribution of TTL (CK19 mRNA copies/μL) shows different median values for each pT stage. Copies: CK19 mRNA copies/μl; Cum (%): cumulative percentage; n: number of cases (PDF 108 kb)
