# Supplementary material for: Molecularly determined total tumour load in lymph nodes of stage I–II colon cancer patients correlates with high-risk factors. A multicentre prospective study
Source: Virchows Arch. 2016 Jul 22;469(4):385–94. doi: 10.1007/s00428-016-1990-1 (PMC5033997; doi:10.1007/s00428-016-1990-1)
Supplement: Supplementary file 1 — Material used for fresh lymph node retrieval. (a) Image of the dissection area, with a thick layer of chopped ice beneath an elevated metallic surface. By placing filter paper above, the fresh LN dissection could be performed on a clean surface. (b) Microcentrifuge tubes were also kept cold by holding them in chopped ice (PDF 160 kb) [file 428_2016_1990_MOESM1_ESM.pdf]

Molecularly determined total tumour load in lymph nodes of stage I-II colon cancer patients correlates with high-risk factors. A multicentre prospective study

Virchows Archiv

Iban Aldecoa, Begoña Atares, Jordi Tarragona, Laia Bernet, Jose Domingo Sardon, Teresa Pereda, Carlos Villar, M Carmen Mendez, Elvira Gonzalez-Obeso, Kepa Elorriaga, Guadalupe Lopez Alonso, Javier Zamora, Nuria Planell, Jose Palacios, Antoni Castells, Xavier Matias-Guiu, Miriam Cuatrecasas

Corresponding author: Miriam Cuatrecasas MD, PhD. Pathology Department (CDB). Escala 3, Planta 5. Hospital Clinic. Villarroel 170, Barcelona 08036. Spain. Tel. +34.93.227.5450 Fax: +34.93.227.5717. e-mail: mcuatrec@clinic.ub.es

Electronic Supplementary Material nº1

Material used for fresh lymph node retrieval

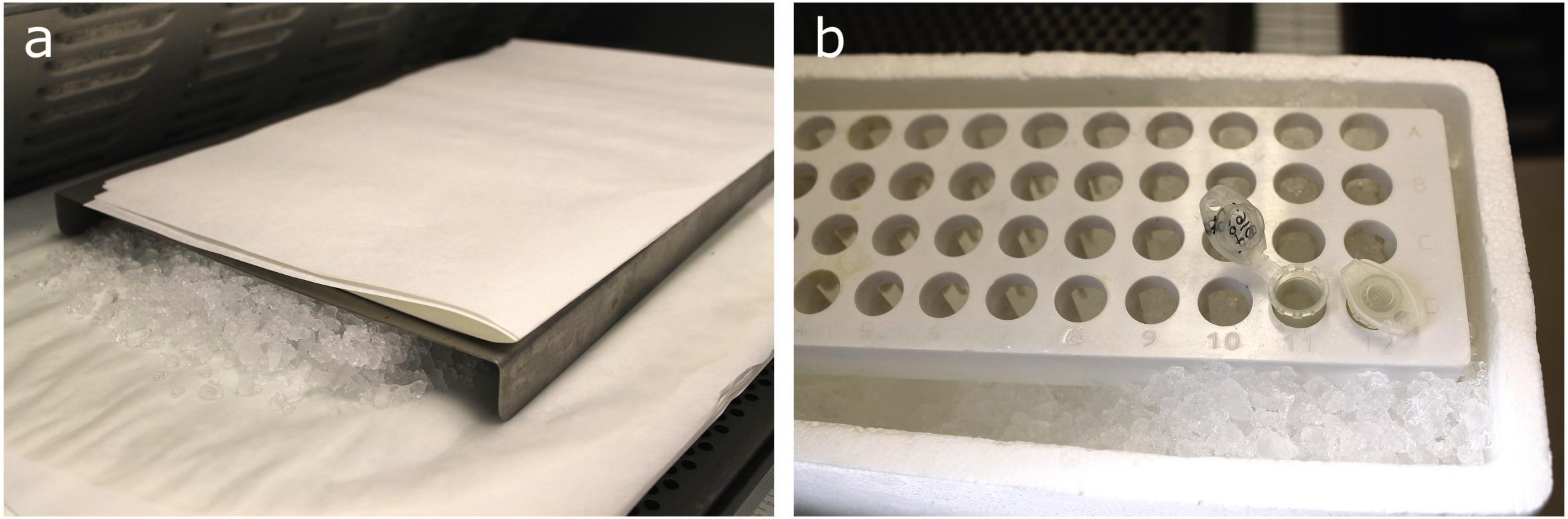

(**a**) Image of the dissection area, with a thick layer of chopped ice beneath an elevated metallic surface. By placing filter paper above, the fresh LN dissection could be performed on a clean surface. (**b**) Microcentrifuge tubes were also kept cold by holding them in chopped ice
