# Supplementary material for: Molecularly determined total tumour load in lymph nodes of stage I–II colon cancer patients correlates with high-risk factors. A multicentre prospective study
Source: Virchows Arch. 2016 Jul 22;469(4):385–94. doi: 10.1007/s00428-016-1990-1 (PMC5033997; doi:10.1007/s00428-016-1990-1)
Supplement: Supplementary file 2 — Case distribution regarding TTL values and pT stage. The distribution of TTL (CK19 mRNA copies/μL) shows different median values for each pT stage. Copies: CK19 mRNA copies/μl; Cum (%): cumulative percentage; n: number of cases (PDF 108 kb) [file 428_2016_1990_MOESM2_ESM.pdf]

Molecularly determined total tumour load in lymph nodes of stage I-II colon cancer patients correlates with high-risk factors. A multicentre prospective study

Virchows Archiv

Iban Aldecoa, Begoña Atares, Jordi Tarragona, Laia Bernet, Jose Domingo Sardon, Teresa Pereda, Carlos Villar, M Carmen Mendez, Elvira Gonzalez-Obeso, Kepa Elorriaga, Guadalupe Lopez Alonso, Javier Zamora, Nuria Planell, Jose Palacios, Antoni Castells, Xavier Matias-Guiu, Miriam Cuatrecasas

Corresponding author: Miriam Cuatrecasas MD, PhD. Pathology Department (CDB). Escala 3, Planta 5. Hospital Clinic. Villarroel 170, Barcelona 08036. Spain. Tel. +34.93.227.5450 Fax: +34.93.227.5717. e-mail: mcuatrec@clinic.ub.es

Electronic Supplementary Material nº2

Case distribution regarding TTL values and pT stage

	Total		pT1		pT2		pT3		pT4a	
**Copies**	**n**	**Cum (%)**	**n**	**Cum (%)**	**n**	**Cum (%)**	**n**	**Cum (%)**	**n**	**Cum (%)**
**250**	1	1,32	0	0,00	1	9,09	0	0,00	0	0,00
**260**	2	3,95	1	4,35	0	9,09	1	2,70	0	0,00
**270**	3	7,89	0	4,35	0	9,09	3	10,81	0	0,00
**320**	1	9,21	0	4,35	0	9,09	1	13,51	0	0,00
**350**	1	10,53	0	4,35	0	9,09	0	13,51	1	20,00
**360**	1	11,84	0	4,35	0	9,09	1	16,22	0	20,00
**400**	1	13,16	1	8,70	0	9,09	0	16,22	0	20,00
**470**	2	15,79	2	17,39	0	9,09	0	16,22	0	20,00
**540**	2	18,42	0	17,39	0	9,09	1	18,92	1	40,00
**620**	4	23,68	3	30,43	0	9,09	1	21,62	0	40,00
**880**	1	25,00	0	30,43	0	9,09	1	24,32	0	40,00
**960**	1	26,32	1	34,78	0	9,09	0	24,32	0	40,00
**1030**	1	27,63	0	34,78	0	9,09	1	27,03	0	40,00
**1180**	3	31,58	1	39,13	2	27,27	0	27,03	0	40,00
**1270**	1	32,89	1	43,48	0	27,27	0	27,03	0	40,00
**1280**	1	34,21	1	47,83	0	27,27	0	27,03	0	40,00
**1320**	1	35,53	0	47,83	0	27,27	1	29,73	0	40,00
**1400**	2	38,16	1	52,17	0	27,27	0	29,73	1	60,00
**1500**	1	39,47	1	56,52	0	27,27	0	29,73	0	60,00
**1540**	1	40,79	0	56,52	0	27,27	1	32,43	0	60,00
**1650**	1	42,11	0	56,52	0	27,27	1	35,14	0	60,00
**1670**	1	43,42	0	56,52	1	36,36	0	35,14	0	60,00
**1790**	1	44,74	0	56,52	1	45,45	0	35,14	0	60,00
**1900**	1	46,05	1	60,87	0	45,45	0	35,14	0	60,00
**1940**	1	47,37	0	60,87	1	54,55	0	35,14	0	60,00
**1980**	1	48,68	0	60,87	0	54,55	1	37,84	0	60,00
**2000**	1	50,00	0	60,87	0	54,55	1	40,54	0	60,00
**2030**	1	51,32	0	60,87	1	63,64	0	40,54	0	60,00
**2200**	1	52,63	1	65,22	0	63,64	0	40,54	0	60,00
**2260**	1	53,95	1	69,57	0	63,64	0	40,54	0	60,00
**2420**	1	55,26	0	69,57	0	63,64	1	43,24	0	60,00
**2460**	1	56,58	0	69,57	0	63,64	0	43,24	1	80,00
**2560**	1	57,89	0	69,57	0	63,64	1	45,95	0	80,00
**3080**	1	59,21	0	69,57	0	63,64	1	48,65	0	80,00
**3140**	1	60,53	0	69,57	0	63,64	1	51,35	0	80,00
**3300**	2	63,16	0	69,57	0	63,64	1	54,05	1	100,00
**3510**	1	64,47	0	69,57	1	72,73	0	54,05	0	100,00
**3600**	1	65,79	1	73,91	0	72,73	0	54,05	0	100,00
**4000**	1	67,11	0	73,91	1	81,82	0	54,05	0	100,00
**4170**	1	68,42	1	78,26	0	81,82	0	54,05	0	100,00

**4250**	1	69,74	1	82,61	0	81,82	0	54,05	0	100,00
**4500**	1	71,05	0	82,61	0	81,82	1	56,76	0	100,00
**5220**	1	72,37	1	86,96	0	81,82	0	56,76	0	100,00
**5600**	1	73,68	0	86,96	0	81,82	1	59,46	0	100,00
**5940**	1	75,00	0	86,96	0	81,82	1	62,16	0	100,00
**7070**	1	76,32	0	86,96	0	81,82	1	64,86	0	100,00
**7120**	1	77,63	0	86,96	0	81,82	1	67,57	0	100,00
**7400**	1	78,95	0	86,96	0	81,82	1	70,27	0	100,00
**7520**	1	80,26	0	86,96	0	81,82	1	72,97	0	100,00
**8110**	1	81,58	0	86,96	0	81,82	1	75,68	0	100,00
**8210**	1	82,89	0	86,96	0	81,82	1	78,38	0	100,00
**8700**	1	84,21	1	91,30	0	81,82	0	78,38	0	100,00
**11530**	1	85,53	1	95,65	0	81,82	0	78,38	0	100,00
**13100**	1	86,84	0	95,65	0	81,82	1	81,08	0	100,00
**15000**	1	88,16	0	95,65	0	81,82	1	83,78	0	100,00
**17280**	1	89,47	1	100,00	0	81,82	0	83,78	0	100,00
**20700**	1	90,79	0	100,00	0	81,82	1	86,49	0	100,00
**21000**	1	92,11	0	100,00	0	81,82	1	89,19	0	100,00
**37180**	1	93,42	0	100,00	1	90,91	0	89,19	0	100,00
**38370**	1	94,74	0	100,00	0	90,91	1	91,89	0	100,00
**47600**	1	96,05	0	100,00	1	100,00	0	91,89	0	100,00
**160130**	1	97,37	0	100,00	0	100,00	1	94,59	0	100,00
**297090**	1	98,68	0	100,00	0	100,00	1	97,30	0	100,00
**297420**	1	100,00	0	100,00	0	100,00	1	100,00	0	100,00

The distribution of TTL (CK19 mRNA copies/µL) shows different median values for each pT stage. Copies: CK19 mRNA copies/µl; Cum (%): cumulative percentage; n: number of cases
